# Group Cognitive Behavioral Analysis System of Psychotherapy (CBASP): A Pilot Study for Bipolar Depression

**DOI:** 10.3389/fpsyt.2020.565681

**Published:** 2020-09-23

**Authors:** Liliane Sayegh, El Hadj Touré, Elisabeth Farquhar, Serge Beaulieu, Suzane Renaud, Soham Rej, Michel Perreault

**Affiliations:** ^1^ Bipolar Disorders Program, Douglas Mental Health University Institute, Montreal, QC, Canada; ^2^ Department of Psychology, McGill University, Montreal, QC, Canada; ^3^ Department of Sociology, University of Montreal, Montreal, QC, Canada; ^4^ Douglas Research Center, Douglas Mental Health University Institute, Montreal, QC, Canada; ^5^ Department of English, Concordia University, Montreal, QC, Canada; ^6^ Department of Psychiatry, McGill University, Montreal, QC, Canada; ^7^ Complex Mood, Comorbid and Personality Disorders Program, Douglas Mental Health University Institute, Montreal, QC, Canada; ^8^ GeriPARTy Research Group, Department of Psychiatry, Jewish General Hospital/Lady Davis Institute, Montreal, QC, Canada

**Keywords:** bipolar depression, unipolar depression, group psychotherapy, severe depression, Cognitive Behavioral Analysis System of Psychotherapy (CBASP)

## Abstract

**Objectives:**

Cognitive Behavioral Analysis System of Psychotherapy (CBASP) is an individually administered treatment model designed specifically for Persistent Depression however bipolar patients have traditionally been excluded from CBASP studies. There is a perception that bipolar depression will be harder to treat and requires a unique psychological approach. This pilot study reports on the feasibility of administering the same 20-week manualized group CBASP therapy with bipolar patients currently in a depressive episode.

**Methods:**

This non-randomized, single-arm prospective pilot study, reports on an *a posteriori* exploration of benefits to bipolar depressed patients (n=26) of the same 20-week group CBASP intervention administered to unipolar depressed patients (n=81). The clinical trial for the initial phase examining benefits of the manualized 20-week group CBASP intervention with unipolar patients was registered with the ISRCTN registry, study ID: ISRCTN95149444. Results reported here include mixed ANOVA analyses, across group treatment models and diagnostic categories. Changes over time in self-reported depressive symptoms (Inventory of Depressive Symptoms -IDS-SR), self-reported social functioning, interpersonal problems and interpersonal dispositions are documented for all patients. An exploratory longitudinal latent class analysis was used to examine patients’ trajectories of improvement in depressive symptoms. Finally, the best predictors of change in reported depressive symptoms were explored with a logistic regression for all patients.

**Results:**

Improvements in depressive symptoms and in social functioning over time were significant for all patients with bipolar patients trending towards a greater improvement in depressive symptoms after controlling for baseline differences. An exploratory Latent Class Analysis identified two different treatment trajectories for the entire sample: 1) moderate to severely depressed patients who improved significantly (49%) and 2) severely depressed patients who did not improve (51%). The best predictors of non-response to group therapy include high baseline problems in social functioning and low rates of self-reported Perceived Improvements in overall health.

**Conclusion:**

Bipolar patients in a depressive episode appear to benefit from the same 20-week group CBASP model designed originally for the treatment of Persistent Depressive Disorder. Bipolar patients seem more easily mobilized both during and outside of group therapy sessions and report more interpersonal confidence and more agency than unipolar depressed patients.

## Introduction

Severe depression is a debilitating illness whether it is associated with a bipolar or unipolar mood disorder and often becomes recurrent and refractory. More than 300 million people are affected by unipolar depression worldwide (WHO 2018) and about 49 million (WHO 2013) have bipolar disorders globally (1, 2). Bipolar disorders follow Major Depressive Disorder as the fifth leading cause of disability-adjusted life years (DALYs) and are the 16^th^ leading cause of years lost to disability worldwide (2). Furthermore, poor psychosocial functioning is a risk factor for illness progression in both bipolar and unipolar mood disorders (3). With bipolar depression being the most difficult to treat and most impairing phase of bipolar disorder (1, 4–6), psychotherapy is often an important adjunct to pharmacotherapy in the treatment of bipolar depression in the context of relapse prevention (6–8). Effective Psychosocial interventions recommended for acute depressive episodes in bipolar disorder include psychoeducation and Cognitive Behavioral Therapy (9) (CBT), Family-focused therapy (10) (FFT) as maintenance treatment with euthymic patients, Interpersonal and Social Rhythm Therapy (1) (IPSRT) and Mindfulness-Based Cognitive Therapy (11) (MBCT) for residual sub-syndromal symptoms. Cognitive Behavioral Analysis System of Psychotherapy [CBASP, (12, 13)] is the only psychotherapy developed to date specifically for the treatment of persistent depression. CBASP is a first line treatment for persistent depression in Europe due to its reported lower drop-out rates and greater tolerability compared to medication and even to other psychological treatments (14, 15). CBASP uses an interpersonal and behavioral paradigm to improve social functioning and help depressed patients break their isolation and improve executive functions (12). It is a highly structured, skills-oriented approach teaching concrete skills to help patients learn interpersonal problem-solving strategies (16). Keller et al. (17) mounted the first long-term, multi-site clinical trial showing the best-yet response rates for chronic depression when individually-administered CBASP and pharmacotherapy are combined. CBASP has been reported on in several meta-analyses on psychotherapy for persistent depression (18–20). In spite of wide heterogeneity between trials being compared, there is consistent evidence of the effectiveness of CBASP as monotherapy for acute depression but even more effectiveness when combined with medication for persistent depression (19, 21).

Group psychotherapy provides a source of social support and rewards as well as exposure to interpersonal interactions and problem-resolution that are needed to improve social functioning in the treatment of chronic depression. In fact studies of psychotherapy for treatment-resistant depression using a group modality were found to have larger effect sizes than individual modalities according to a recent meta-analysis and meta-regression (22). The feasibility of a maximum of 10 sessions of CBASP group therapy adapted for chronically depressed unipolar inpatients was assessed in a large multicenter study by Sabass et al. (23). The concept of acceptability was measured by self-report questionnaires completed by patients and therapists separately. In addition to significant improvements in clinician-rated depression scores, in self-reported depressive symptoms and in quality of life, results are highly favorable, according to the authors, with regards to acceptability and clinical benefits of group CBASP, considering the lower number of group sessions. Guhn et al. (24) assessed a 12-week multimodal CBASP treatment adapted for inpatients with Persistent Depressive Disorder which included a total of 26 individual and group CBASP sessions with 4 weeks of post-treatment outpatient group CBASP sessions. The group sessions comprised of the same CBASP adaptation used in the previous study by Sabass et al. however the setting in this study was a general psychiatric ward. Guhn et al. (24) report significant treatment response at post-treatment and significant but lower response rates at 6-month follow-up with regards to clinician-rated and to a lesser extent self-rated depressive symptoms. They confirm that their sample of more severely depressed patients than previously reported in a meta-analysis of psychotherapy for chronic depression (21), may have benefitted from an extended duration of treatment to consolidate improvements. Improvements in depressive symptoms were also associated with reduced interpersonal distress.

In a prospective, bi-center, randomized controlled trial, Michalak et al. (25) compared a group adaptation of CBASP plus treatment as usual (TAU) to Mindfulness-Based Cognitive Therapy (MBCT) plus TAU, to TAU alone in a sample of patients with chronic major depression or Persistent Depressive Disorder. Results revealed a large effect size for improvements in clinician-rated depressive symptoms with eight sessions of group CBASP in addition to treatment as usual for the entire sample while group MBCT was effective in one treatment site more than the other. Comparisons between CBASP and MBCT were complicated by between-site differences discussed by the authors.

There is no published study documenting the effectiveness of group CBASP with bipolar depression. Although McCullough designed and evaluated the effectiveness of CBASP with patients suffering from unipolar persistent depression (17) and a bipolar disorder diagnosis constituted an exclusion criterion in all studies on the effectiveness of CBASP, there is no stipulated contraindication for the use of CBASP for bipolar depression. Furthermore, the same DSM-5 criteria and symptom duration are used to diagnose a major depressive episode in either diagnostic category. Perhaps bipolar patients have been excluded from CBASP treatment due to concerns that patients may switch unexpectedly into hypomania during treatment or to concerns that patients may have cognitive difficulties that make it difficult for them to benefit from cognitive restructuring exercises. The rationale behind staging models for psychiatric disorders sometimes suggests that Bipolar Disorders are more difficult to treat or require different therapeutic interventions or models in relation to the progressive nature of the illness (26–28) Therefore, it remains unknown whether group CBASP can also be beneficial for bipolar depression, a mood state that is often more long-lasting than hypomanic states.

The first author (LS) manualized the group adaptation of CBASP (29, 30) for persistent depression used in this study. In a pilot study administering this group CBASP adaptation over 12 weekly sessions with a sample of unipolar depressed outpatients, it was found to be beneficial in reducing self-reported depressive symptoms and improving self-reported social adjustment and interpersonal self-efficacy (31). However, twelve sessions were found to be insufficient to reach community-based levels of social functioning. A second pilot study was carried out seeking to verify the benefits of increasing this manualized group CBASP adaptation from 12 to 20 weeks considering previous findings of insufficient duration (31). The choice of 20 weeks was based on findings of a meta-analysis of the effectiveness of psychotherapy trials for chronic major depression and dysthymia suggesting 18 treatment sessions to be optimal (21). The second pilot study kept the same outcome measures of self-reported depressive symptoms and social functioning. A sample of unipolar severely depressed patients were non-randomly assigned in a sequential manner to either 20 weeks of manualized group CBASP treatment or to 20 weeks of a manualized group adaptation of Behavioral Activation for depression, also known to be effective in treating depression. Some results of this comparison have been previously published (32). The second pilot study examining the benefits of an extended 20-week group CBASP treatment for unipolar severely depressed patients was registered as a clinical trial with the ISRCTN registry, study ID: ISRCTN95149444.

An exploratory a posteriori phase of this second pilot study, reported in this article, involved administering this group CBASP treatment with bipolar patients in a current depressive episode. This idea emerged largely out of necessity to provide some psychotherapy treatment to severely depressed bipolar patients. This phase followed the completion of data collection with unipolar patients. In this first feasibility, pilot study of group CBASP including bipolar depressed patients, the main objective is to assess whether these patients will benefit from the same 20-weeks of manualized group CBASP treatment, with regards to self-reported improvements in depressive symptoms and in social functioning, as is administered with unipolar depressed patients. The study will also assess whether differences (if any) between patients with bipolar depression or unipolar depression persist after controlling for differences in baseline characteristics. Analyses will explore the nature of the relationship between trajectories of improvement in depressive symptoms and changes in social functioning throughout group therapy for both bipolar and unipolar depressed patients. The best predictors of symptomatic improvements will be examined for all patients. In addition, using a latent class analysis, trajectories of improvements in depression will be explored as well as any associated characteristics.

## Materials and Methods

### Study Design and Participants

**Figure 1:**
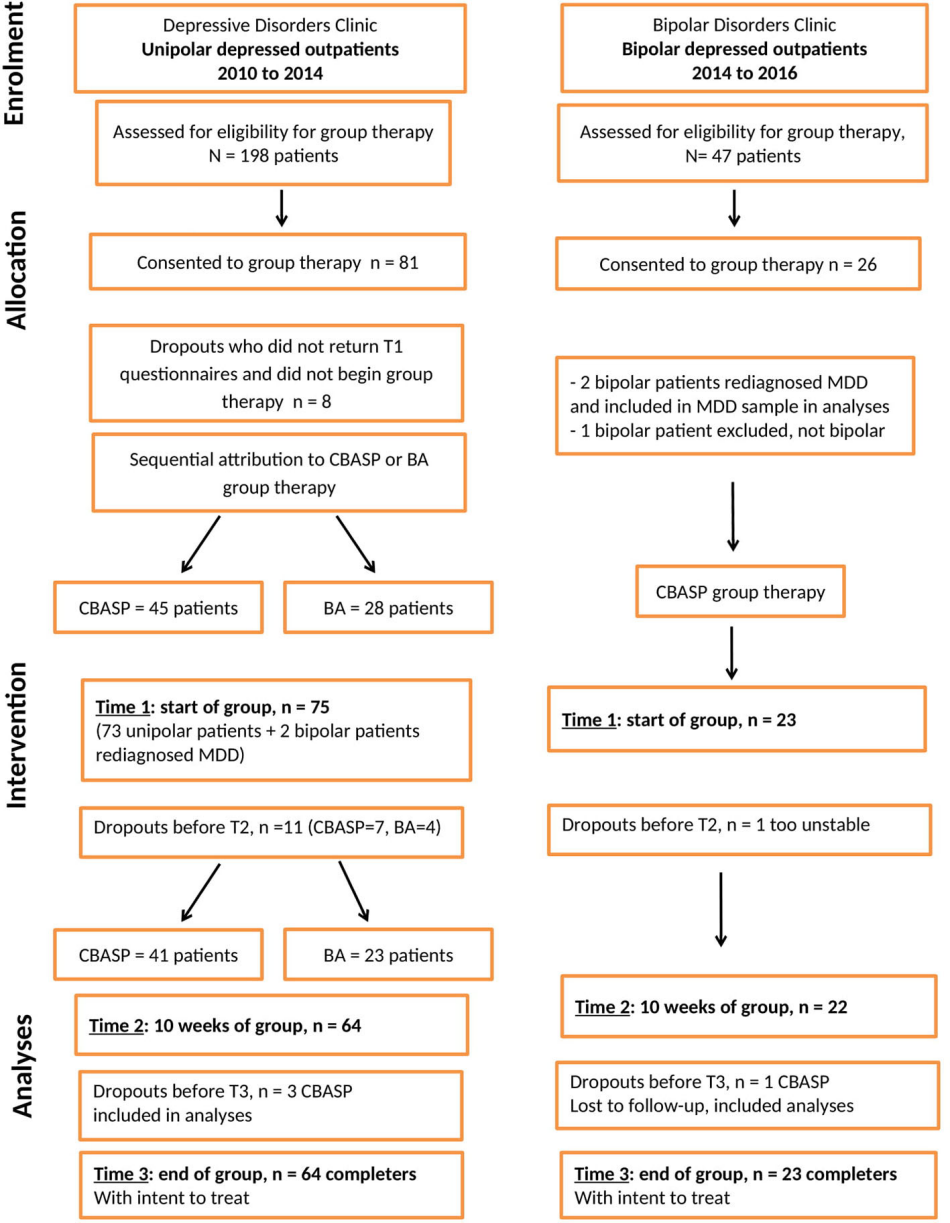
CONSORT flow chart.

This pilot study is a non-randomized, single-arm prospective study. All participants were adult outpatients at the Douglas Mental Health University Institute, Montreal, Quebec, Canada, a specialized, tertiary-care teaching/research psychiatric hospital. Patients with a bipolar depression were treated at the Bipolar Disorders Clinic and patients with a unipolar depression were treated at the Depressive Disorders Clinic. Similar administrative procedures are present in both clinics with regards to admission of patients, semi-structured psychiatric assessments and psychological services available. Participants were included in the study in a sequential manner based on the time of their referral, between 2010 and 2016. The enrolment of participants in both clinics is described in the CONSORT flow-chart in **Figure 1**. Therapy groups consisted entirely of bipolar or of unipolar patients since group therapies were held in each respective clinic where the treating psychiatrists and the patients’ charts were situated. Groups were not comprised of patients from both clinics together due to an administrative directive at the time. All participants underwent a comprehensive DSM-IV-TR-based psychiatric evaluation required for admission into the clinics, carried out by their treating psychiatrist. Unipolar patients all received their psychiatric diagnosis before the DSM-5 introduced the diagnosis of Persistent Depressive Disorder to replace several categories for chronic depression. These diagnoses have not been revised for this study. Pharmacological treatments provided for each patient followed clinical guidelines for treatment algorithms developed by the Canadian Network for Mood and Anxiety Treatments (CANMAT) for patients with Major Depressive Disorders (33) and by CANMAT as well as the International Society for Bipolar Disorders (ISBD) regarding patients with a Bipolar Disorder (1). Recommendations for concurrent psychosocial therapies are offered, according to CANMAT, as part of best management practices for Bipolar Disorders (8) and for unipolar Major Depressive Disorder (34). Inclusion criteria were patients between ages 18 and 65 with a DSM-IV-TR primary diagnosis of Major Depressive Disorder (MDD), unipolar, or a diagnosis of Bipolar Disorder Type I or Type II, current episode depression, or schizoaffective bipolar type. In addition to primary diagnoses, a second comorbid Axis-I diagnosis was recorded for each patient. Exclusion criteria included the following: psychosis or psychotic symptoms during group therapy, an acute manic or hypomanic episode, a primary diagnosis for any of: anxiety disorders, schizophrenia, acute substance abuse disorder, eating disorder. Patients with a debilitating or unstable medical diagnosis were also excluded. Patients, who were at high risk for suicide at the start of group therapy, including acute suicidal ideation, intent or suicide attempt, were excluded in favor of an individual intervention. This study was approved by the Douglas Institute’s Research Ethics Board (REB Protocol 10/19).

### Procedures

Patients were referred to group therapy by their treating psychiatrist for a concurrent psychological intervention to their pharmacological treatment due to the severity of their depressive symptoms. Patients from the Depressive Disorders Clinic were met individually by the CBASP-certified psychologist and informed of the benefits of CBASP and BA in a group format for chronic depression and of the option to participate in the study of their benefits. In accordance with the declaration of Helsinki, patients who accepted to participate in group therapy signed a consent form informing them of the research study and of its impact. Patients who refused group therapy were offered other psychological interventions in an individual modality as they most often refused a group format. A few of the patients who dropped out after giving their consent may have done so in rejection of the group format which they are not always comfortable to admit to. Baseline demographic or clinical information on these dropout cases were included in analyses to assess for potential differences with patients who remained in group therapy. However, personality characteristics, not assessed in this pilot study, may contribute to patients’ preferences with regards to treatment modality. Following their informed consent to undergo group therapy, patients from the Depressive Disorders Clinic were assigned to either CBASP or BA group therapy in a sequential manner by constituting one group of six patients for CBASP or BA and then another group of six patients for the other treatment model, etc. Patients were taken from the waiting list and assessed for eligibility for group therapy when the next CBASP or BA group was being constituted. This followed administrative procedures regarding the use of waiting lists for requests for psychotherapy services in this public mental health clinic. Patients did not receive any other psychological treatments while on the waiting list, since group therapy had been recommended, nor during participation in group therapy in order not to confound treatment effects. Medical follow-ups are provided to all patients admitted to the clinic while on a waiting list for psychotherapy.

Patients from the Bipolar Disorders Clinic were offered CBASP group therapy due to a demand for psychological treatment options for the depressive phase of the disorder. Therefore an *a posteriori* exploration of benefits of CBASP for bipolar depression seemed a logical follow-up of this initiative. All bipolar patients participated in CBASP group therapy after all unipolar patients had completed their participation in the study. Bipolar patients also gave their informed consent to complete questionnaires aimed at assessing the benefits of CBASP group therapy.

All patients, unipolar and bipolar, were met for two individual sessions prior to beginning group treatment in order to determine the interpersonal goals they would focus on during group therapy. The group treatment comprised one 2-hour session each week for 20 consecutive weeks, held in each unipolar and bipolar clinics separately. The group CBASP manual adapted by the first author (LS) (29, 30) is based on McCullough’s CBASP individual modality (12, 13). The group BA manual developed by Lejuez et al. (35) was adapted by the first author (LS) to accommodate the 20-week group treatment protocol. Each group had a maximum of six patients, with the median and modal group size being five patients. CBASP groups were conducted by a CBASP-certified senior clinical psychologist with a clinical psychology graduate student as co-therapist, receiving training in CBASP or by two CBASP-trained psychology graduate students, all supervised by a CBASP-certified senior clinical psychologist. All BA groups were conducted by either a clinical psychologist or by experienced psychotherapists on staff or by nurse clinicians with a clinical psychology graduate student as co-therapist, also trained and supervised by a clinical psychologist.

All patients received routine medical appointments with their psychiatrist throughout group therapy, examining symptomatology and required minimal changes to their long-term medication regimen. When patients needed to be hospitalized during group therapy, their ongoing participation in group sessions was individually determined. This means that patients could continue attending group sessions during hospitalization and did not drop-out nor were they excluded from participating in group therapy, unless a patient requested to stop group therapy. Patients who completed the initial assessment but subsequently dropped out were included in the current analyses in order to determine whether any characteristics are associated with dropouts.

### Patient Selection

A total of 107 outpatients (bipolar n=26, unipolar n=81) admitted into the clinics sequentially accepted to participate in the study. **Figure 1** displays in a CONSORT flow chart enrolment, allocation and intervention stages with dropout cases identified at each level. One bipolar patient, whose primary diagnosis of Bipolar Disorder was changed, was excluded from the study and two bipolar patients had their diagnosis revised to unipolar depression (MDD) and were included in the unipolar sample. One unipolar patient was excluded from analyses due to ongoing psychotic symptoms. The following patients with complete information on clinical and outcome measures, with attrition accounted for, included in analyses at the three assessment periods consisted of: 98 patients at time 1 (23 bipolar, 75 unipolar), 86 patients at time 2 (22 bipolar, 64 unipolar) and 82 patients at time 3 (21 bipolar, 61 unipolar). Patients who did not complete the 20 weeks of group therapy, but attended more than half of the sessions, were included in the analyses using the Intent to Treat principle, as indicated in **Figure 1**. No significant differences were found for both samples between drop-outs due to attrition and patients who completed the 20 weeks of treatment on all demographic and clinical variables. Participants (total N=104) included in the analyses reported below, consisted of 23 bipolar patients in group CBASP, 41 unipolar patients in group CBASP and 23 unipolar patients in group BA. Analyses included demographic and baseline information for non-completers in order to test for differences.

### Group Treatments

Cognitive Behavioral Analysis System of Psychotherapy (CBASP), developed by McCullough (12, 13), is the only psychotherapy developed specifically to treat the chronically depressed patient. Based on contemporary learning theory, its primary goal is to connect the patient perceptually to others (the environment) so that others can begin to inform/influence the behaviour of the patient in positive ways. CBASP is based on a Person X Environment Causal Determinant Model of Behavior and promotes the acquisition of stimulus learning (through the therapeutic and other more adaptive relationships) and response learning (acquiring more adaptive coping behaviours to reduce interpersonal avoidance and increase positive reinforcements) (36).

Both group treatment adaptations, CBASP and BA, comprised two modules. The first module introduces a behavioural activation exercise using an activity calendar and graded task assignments to promote a more active life style. In the case of the CBASP group treatment, the second module introduced the CBASP model with its components including the Situational Analysis, use of the Transference Hypothesis and Interpersonal Discrimination Exercise. The Interpersonal Circumplex is used to demonstrate how complementary interpersonal behaviours result in satisfactory exchanges and how interpersonal motives and goals drive our interpersonal interactions. The Situational Analysis (SA) is used to teach participants the consequences of their interpersonal behaviours. Social skills are also practiced during the SA with participants carrying out role plays together.

The group Behavioral Activation manual used was developed by Lejuez et al. (35, 37) and is based on behavioural principles that examine mechanisms of behavioural change. The goal of this treatment is to gradually increase the frequency of targeted healthy behaviours by increasing the relative value of such behaviours for the individual. This treatment model suggests that the relative frequency of depressed behaviours, compared to non-depressed behaviours (that is all other types of behaviours), is proportional to the relative value of reinforcement obtained for depressed behaviours compared to non-depressed behaviours (37). As explained above, the first module of the group BA treatment introduced a behavioral activation exercise to promote an active life style. The second module introduced the initial stage of BA treatment consisting in assessing the function of depressed behaviours, whether these are maintained by (a) an absence of reinforcement for non-depressed behaviours, (b) reinforcement for depressed behaviours, or (c) some combination of the two. The rest of the group sessions consisted in patients reviewing and setting short-term goals with regards to various aspects of their life (social, leisure, work, personal) and determining how to realize these goals with the use of graded tasks which they reported on each week. BA group sessions did not focus on interpersonal problems or strategies to resolve them.

### Primary Outcome Measure

All participants in the study were assessed three times at approximately 10-week intervals: at the beginning of group treatment (baseline T1: time 1), at the 10^th^ week of treatment (mid-treatment T2: time 2), and at the 20th week of treatment (termination T3: time 3). The outcome measure used was changes in depressive symptoms recorded with the Inventory of Depressive Symptoms, Self-Report [IDS-SR, (38)]. This is a 30-item measure of symptoms of depression experienced during the previous week. Items are scored on a 0 to 3 scale, with higher scores reflecting more severe depression. Rush et al. (38) report Cronbach’s alpha coefficients of internal consistency of .77 for a sample of symptomatic depressed patients. The authors also report good discriminant validity of the IDS-SR between symptomatic and euthymic patients with MDD and report that the IDS-SR is equivalent to the Hamilton Rating Scale for Depression (39) in detecting symptom change during an acute treatment phase. Rush et al. (40) also report good concurrent and discriminant validity for the IDS-SR and sensitivity to change in patients with major depressive and bipolar disorders. Trivedi et al. (41) reported good psychometric properties for the IDS-SR with a public sector psychiatric outpatient sample of patients with Major Depressive Disorder (MDD) or Bipolar Disorder (BD). In addition, internal consistency scores for the IDS-SR were 0.92 for patients with MDD and 0.89 for the clinician-rated IDS with bipolar patients.

#### Baseline Characteristics

The same self-report measures were used with both samples of bipolar and unipolar patients. Baseline characteristics were assessed on demographic, clinical and social domains of functioning as well as for perception of improvement with treatment received. Demographic variables include age, gender, marital status, employment status, while clinical variables of interest include symptom severity, duration of current depressive episode, total number of depressive episodes and co-morbid psychiatric diagnoses.

### Secondary Outcome Measures

#### Social Adjustment Scale—Self-Report

The Social Adjustment Scale, self-report [SAS-SR, (42)] is a 54-item self-report questionnaire assessing instrumental and expressive role performance over the past two weeks. Six major areas of functioning are covered: work (paid worker, unpaid homemaker or student); social and leisure activities; relationships with extended family; role as a marital partner; parental role; and role within the family unit, including perceptions about one’s economic situations. Each question is rated on a 5-point scale with higher scores indicating more impairment. A mean for each role category is obtained as well as an overall adjustment score. The SAS-SR has a good internal consistency coefficient of 0.74 and a good test-retest reliability coefficient of 0.78 over a period of two weeks. The Alpha coefficient for the current sample of depressed patients is 0.71. Patients’ mean total SAS-SR scores at Time 1 in this study’s sample, are comparable to the scores reported by Weissman et al. (43) for patients in acute depression.

#### Perceived Stress Scale

The Perceived Stress Scale (PSS, [(44)] is originally a 14-item scale designed to measure the degree to which situations in one’s life are appraised as stressful. Items were designed to tap how unpredictable, uncontrollable and overloaded respondents find their lives with high scores indicating more perceived stress. The shorter 10-item version, with an alpha coefficient for internal reliability of 0.78, is used in the present study (45).

#### The Coping Inventory for Stressful Situations

The Coping Inventory for Stressful Situations [CISS, (46)] is a 48-item, self-report questionnaire using a 5-point Likert-type rating scale ranging from (1) “not at all” to (5) “very much” with high scores indicating greater use of coping strategies reported. The CISS is comprised of three coping dimensions: Task, Emotion and Avoidance-oriented coping strategies. There are two subscales for the Avoidance-Oriented scale: Distraction and Social Diversion. High alpha reliability coefficients for internal consistency for a psychiatric normative group range from 0.69 to 0.91. Test-retest reliabilities were moderate to high for the Task and Emotion scales (0.68 to 0.73) and moderate for the Avoidance scale (0.51 to 0.60). Good construct validity was found when comparing the CISS with the Ways of Coping Questionnaire [WCQ, (47, 48)], in the directions predicted.

#### Perceptions of Improvement Questionnaire

The Perceptions of Improvement Questionnaire (PIQ) is a self-report questionnaire measuring patients’ perceptions of improvements of their physical and mental health symptoms (49). It was administered at 10 and 20 weeks of group therapy. Patients rate on a 4-point rating scale, ranging from “worse than before” to “much better than before”, the extent to which they perceive improvement in 20 areas of their life, since the start of group therapy. High scores indicate more reported improvements. In a study with 232 participants in a methadone maintenance program, a factor analysis of this scale generated three main factors, accounting for 60.1% of the variance, including emotional health, social relations and physical health (49). Internal consistency coefficient for the overall scale is 0.91 and for the three subscales were 0.91 for “emotional health”, 0.79 for “social relations”, and 0.79 for “physical health”. Cronbach’s alpha coefficients for the current study sample are 0.90, 0.78, 0.68 and 0.63 for the overall scale, emotional health, social relations and physical health respectively.

#### Circumplex Scales of Interpersonal Problems, Values and Efficacy

The 32-item circumplex version of the Inventory of Interpersonal Problems used in this study [IIP-32, (50)] is a self-report questionnaire assessing interpersonal difficulties and distress generated. Respondents rate 4 items, for each of 8 octants in the circumplex, on 0 (not at all) to 4 (extremely) scales with high scores indicating high interpersonal distress. The internal consistency for the IIP-32 is high with reliability coefficients ranging from 0.68 to 0.93. The Circumplex Scales of Interpersonal Values [CSIV, (51)] is a 64-item self-report measure of interpersonal goals or values (8 items for each of 8 octants) for which respondents rate the importance for themselves (on a scale from 0, not important, to 4, extremely important). The scale demonstrates very good internal consistency for the eight octants of the circumplex, with a Cronbach’s alpha ranging from 0.76 to 0.86. The Circumplex Scales of Interpersonal Efficacy [CSIE, (52)] is a 32-item self-report measure of individuals’ confidence in their ability to perform interpersonal behaviours successfully. Respondents were asked to rate (on a 0, not at all confident, to 10, absolutely confident) 4 interpersonal actions for each of 8 octants. Responses were transformed to 0-to-4 scales to make them comparable to the IIP and CSIV scales. In the current study respondents were asked to think of the group therapy setting as an example of interpersonal situations they were asked to rate for the CSIV and CSIE. The scales of the CSIE have been shown to have internal consistency (Cronbach alphas ranging from 0.66 to 0.83 for each of the 8 octants). Satisfactory Cronbach alphas for a similar sample of persistently depressed outpatients, taken from the same mental health institute as the current study sample, were previously reported for these three circumplex measures (53).

A structural summary approach for calculating scores derived from these circumplex scales yields vector lengths for each scale which can represent indicators of a person’s interpersonal style (54). Longer vector lengths on any scale suggest a more limited interpersonal repertoire with high scores in one particular region of the circumplex but low scores in the opposite regions. Shorter vector lengths suggest an equal distribution of scores on opposite sides of the circumplex. Therefore, individuals with personality dispositions in all regions of the circumplex (shorter vector lengths) can be described as more interpersonally flexible and more able to adapt to the demands of a situation. Whereas individuals with a more limited interpersonal repertoire may only be able to express the same set of behaviors even if these are inappropriate to the situation (54).

#### Weekly Journal

All patients were asked to complete a Weekly Journal at the beginning of each of the 20 group sessions, consisting of 20 items, on a 5-point Likert-type scale ranging from “not at all” to “every day.” Six items assessed *behavioral activation* (e.g., “I have completed my household chores and/or professional/student work”). Six items assessed *depressive symptoms* similar to DSM criteria (e.g., “I have been in a sad, depressed mood”). Eight items assessed interpersonal *self-efficacy* (CSIE), one item for each of the 8 CSIE octants; (eg., “This week in the group I can be helpful, I can take an active part in the group, I can ease the pain of others, and I can understand their feelings”). Whereas the *self-efficacy* items referred to expectations for the coming week, the *activation* and *depression* items referred to experiences over the preceding week. Cronbach’s Alpha coefficients of reliability obtained for this study sample for each of these three subscales are 0.78, 0.81 and 0.60 for BA, DSM symptoms and CSIE, respectively.

### Data Analyses

Using SPSS 25 (Statistical Package for the Social Sciences), a series of chi square and ANOVAs were used to compare bipolar patients who underwent group CBASP, unipolar patients who underwent group CBASP, and unipolar patients who underwent group BA using frequencies/means for demographic and clinical baseline measures. Cramer’s V, Cohen’s D and Eta Square were used as association measures to assess effect sizes of significant differences between groups.

Measures of depressive symptoms over time (T1, T2, and T3) were compared between the two diagnostic groups using a mixed multivariate ANOVA (repeated measures and groups analysis), controlling for covariates which comprised total number of depressive episodes and comorbid diagnoses (SPSS).

Then, a mixed ANOVA (repeated measures by diagnostic groups) was performed to assess changes in each clinical and social measure (SAS, CISS, PSS, IIP-32, CSIE, CSIV, PIQ, and Weekly Journal) over time across bipolar and unipolar patients. Partial Eta square is used as an association measure to assess effect size of main or interaction effects.

Mplus 8.1 (55) was used to perform an exploratory longitudinal latent class analysis to identify subgroups of patients’ trajectories of improvement in depressive symptoms. The structural software maximizes the information available in the data, concerning IDS-SR, so as to complete the missing repeated measurements (n=98) for patients who did not complete 20 weeks of treatment. To select the optimal solution of latent classes of depressive symptoms over time, we used at least three statistical indexes: Bayesian Information Criterion (BIC), Entropy, and Lo Mendel Rubin (LMR) adjusted test. The best solution chosen is the model with the lowest BIC, an entropy or rate of classification larger than 0.70, to which an additional class does not improve statistically the retained model after an LMR test. For this exploratory analysis, the selected model must be substantively meaningful for robustness.

Two optimal latent classes obtained were used to perform a mixed ANOVA in order to confirm whether the latent class trajectories are significantly different with regards to depressive symptoms over time. Finally, multiple comparisons were performed to validate statistically and clinically these two latent classes. We conducted several t-tests and chi-square tests to assess differences between these latent classes with regards to demographic and clinical characteristics.

A Logistic Regression Analysis was carried out to identify, among baseline social functioning variables the ones that best predict the trajectory group of patients who benefit most from group therapy with regards to self-reported improvements in depressive symptoms. Given the relatively small sample size (n=104), only the most important variables are included in the multivariate model to assess class membership. These variables must be significant in the bivariate analysis.

## Results

Demographic and baseline information on the sample (N=104) are outlined in **Tables 1A**, **B**. At baseline, patients with bipolar depression (n=23) did not differ significantly from those with unipolar depression who received group CBASP (n=41) or from unipolar patients who received group BA (n=23) with regards to most demographic, clinical and social functioning characteristics. However, bipolar depressed patients had a greater total number of depressive episodes (4.8 bipolar vs. 2.9 unipolar, t=4.33, p≤.001, Cohen’s D=0.88), more comorbid psychiatric diagnoses (primarily Anxiety Disorders and Substance Abuse Disorders in remission, 87% bipolar vs. 28% unipolar, Chi^2 =^ 25.3, p ≤ 0.001, Cramer’s V=0.49), and the week prior to beginning group therapy bipolar patients reported in the Weekly Journal lower levels of behavioral activation on a daily basis (3.7 bipolar vs. 8.3 unipolar, t=3.34, p ≤ 0.001, Cohen’s D=0.76) but fewer self-reported depressive mood symptoms (4.8 bipolar vs. 13.3 unipolar, t=4.88, p ≤ 0.001, Cohen’s D=1.11) compared to unipolar depressed patients. All patients had similar IDS-SR depression severity at baseline.

**Table 1A:** Participant characteristics by treatment groups: Comparison of frequencies.

T1-Characteristics	Treatment groups					
	Bipolar CBASP	Unipolar CBASP	Unipolar BA	All sample	Chi-2	Cramer V
n	23	41	23	87		
Gender						
Female	11 (48)	20 (49)	17 (74)	48 (55)	4.44	–
Male	12 (52)	21 (51)	6 (26)	39 (45)		
Marital status					7.87	–
Maried	4 (18)	12 (29)	11 (48)	27 (31)		
Single/divorced	12 (52)	21 (51)	11 (48)	44 (51)		
In a relationship	7 (30)	8 (20)	1 (4)	16 (18)		
Employment status					11.48	–
Employed	3 (13)	8 (19)	1 (4)	12 (14)		
Unemployed	7 (30)	16 (39)	16 (70)	39 (45)		
Sick leave	10 (44)	15 (37)	6 (26)	31 (36)		
Student/retired	3 (13)	2 (5)	0 (0)	5 (6)		
Psychiatric Comorbidity					20.48***	.49
Yes	20 (87)	12 (29)	9 (39)	41 (47)		
No	3 (13)	29 (71)	14 (61)	46 (53)		
Type of comorbidity					–	–
Anxiety only	5 (23)	2 (5)	1 (4)	8 (9)		
Anxiety & other	6 (27)	1 (2)	0 (0)	7 (8)		
Alcohol	0 (0)	1 (2)	0 (0)	1 (1)		
Alcohol in remission	1 (4)	2 (5)	2 (9)	5 (6)		
Substance abuse	1 (4)	4 (10)	6 (26)	11 (13)		
Substance in remission	5 (23)	0 (0)	0 (0)	5 (6)		
Situational disorder	0 (0)	1 (2)	0 (0)	1 (1)		
Gambling	1 (4)	0 (0)	0 (0)	1 (1)		
No comorbidity	3 (14)	29 (73)	14 (61)	46 (54)		
Type of diagnostic					–	–
MDD	0 (0)	20 (49)	15 (65)	35 (40)		
MDD recurring	0 (0)	18 (44)	8 (35)	26 (30)		
MDD single	0 (0)	2 (5)	0 (0)	2 (2)		
Double depression	0 (0)	1 (2)	0 (0)	1 (1)		
Bipolar Type I	8 (35)	0 (0)	0 (0)	8 (9)		
Bipolar Type II	13 (57)	0 (0)	0 (0)	13 (15)		
Schizoaffective bipolar	1 (4)	0 (0)	0 (0)	1 (1)		
Bipolar rapid cycles	1 (4)	0 (0)	0 (0)	1 (1)		

Entries are frequencies (with percentages in parenthesis). Percentages are calculated among the diagnostic variable. The Cramer’s V statistic is a measure of association. ***p ≤ 0.001 (two tailed-test). For type of comorbidity and type of diagnostic variables, no chi-square test is calculated because there is zero in the cellules.

**Table 1B:** Participant characteristics by treatment groups: Comparison of means.

T1-Characteristics	Treatment groups						
	Bipolar CBASP	Unipolar CBASP	Unipolar BA		All sample	F	Eta square
n	23	41	23		104		
Age	48.5 (11.1)	43.4 (11.1)	48.3 (8.5)		46.0 (10.6)	2.51	–
Duration of current episodes (months)	20.6 (16.3)	28.8 (26.5)	24.7 (19.7)		25.5 (22.5)	0.99	–
Total depressive episodes	4.81 (3.23)	3.10 (1.37)	2.70 (0.82)		3.43 (2.09)	7.88***	0.16
IDS-SR	37.3 (10.9)	39.0 (12.5)	36.1 (13.9)		37.8 (12.4)		–
SAS mean	2.46 (0.55)	2.66 (0.58)	2.58 (0.44)		2.59 (0.54)	0.97	–
Work Role	2.97 (1.15)	3.22 (1.27)	3.57 (1.29)		3.25 (1.25)	1.30	–
Social leisure	3.13 (0.82)	2.97 (0.59)	2.81 (0.81)		2.97 (0.72)	1.22	–
Extended family	2.14 (0.56)	2.27 (0.53)	2.18 (0.61)		2.21 (0.56)	0.43	–
Primary relation	2.12 (0.60)	2.54 (0.67)	2.32 (0.50)		2.37 (0.62)	1.99	–
Parental role	1.86 (0.48)	2.05 (0.63)	1.83 (0.58)		1.96 (0.57)	0.42	–
Family unit	1.93 (0.76)	2.43 (1.10)	2.02 (1.06)		2.19 (1.03)	2.23	–
Vector Length CSIE	3.31 (1.79)	3.51 (1.62)	4.05 (1.47)		3.58 (1.64)		–
Vector Length CSIV	1.20 (0.61)	1.24 (0.53)	1.20 (0.39)		1.22 (0.52)	0.17	–
Vector Length IIP	1.90 (0.95)	1.81 (0.84)	2.14 (0.69)		1.93 (0.83)	0.02	–
CISS coping							
Task oriented	42.9 (11.7)	44.5 (9.7)	41.8 (9.45)		43.4 (10.2)	0.55	–
Emotion oriented	53.2 (8.4)	54.1 (8.3)	51.3 (9.84)		53.1 (8.7)	0.74	–
Avoidance oriented	36.8 (9.9)	38.7 (8.0)	37.4 (8.23)		37.9 (8.5)	0.40	–
Distraction	19.3 (6.4)	19.8 (4.7)	19.6 (4.42)		19.6 (5.1)	0.08	–
Social diversion	10.6 (3.5)	12.7 (4.9)	11.0 (3.76)		11.7 (4.3)	2.14	–
PSS stress	26.5 (6.0)	26.7 (5.7)	26.04 (3.83)		26.5 (5.3)	0.10	
T2-Perceived efficacy	29.3 (7.8)	26.2 (8.11)	27.04 (5.90)		27.2 (7.5)	1.20	–
Emotional health	8.1 (2.4)	7.2 (2.6)	6.91 (2.31)		7.4 (2.5)	1.52	–
Social relations	5.2 (1.6)	4.9 (1.8)	4.60 (1.5)		4.9 (1.7)	0.76	–
Physical health	6.5 (2.4)	5.6 (2.0)	5.65 (1.5)		5.8 (2.0)	1.54	–
Weekly Journal							
Behavioral activation	3.74 (5.83)	8.19 (5.74)	9.74 (4.41)	7.20 (5.9)		7.1**	0.17
Depressive symptoms	4.78 (7.46)	13.06 (7.02)	14.58 (5.82)	10.9 (7.9)		13.4***	0.27
CSIE	5.61 (8.34)	13.5 (7.16)	15.42 (2.82)	11.5 (7.8)		13.2***	0.27

Entries are means (with standard deviation in parenthesis). The Cohen’s statistic is a measure of effect size: 0.20 = Small effect; 0.50 = Medium effect; 0.80 = Large effect. ***p ≤ 0.001 (two tailed-test) **p ≤ .01.

Both bipolar and unipolar patients had a similar duration of the current depressive episode for which they were in group therapy, with a slightly higher duration for unipolar patients, the mean duration being 25 months for the entire sample. Bipolar patients had an average of almost two hypo/manic episodes (SD=1.82).

The absence of significant differences between CBASP and BA group therapies on all baseline measures allowed us to combine results for all unipolar patients and to focus on trajectories of change in depressive symptoms and social functioning in bipolar and unipolar patients.

**Figure 2:**
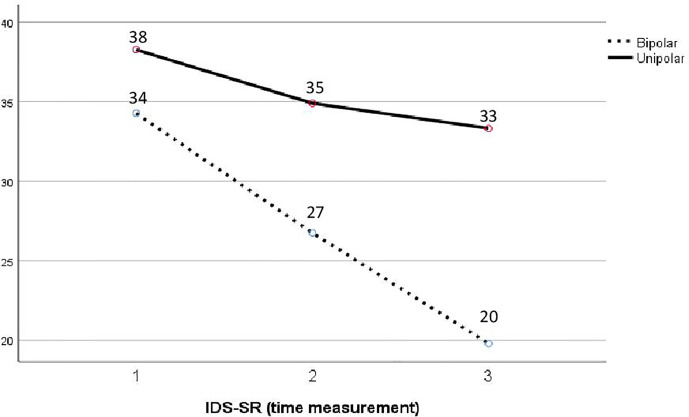
Mean trajectories of depressive symptoms (IDS-SR) over time for diagnostic groups, controlling for psychiatric comorbidity and total number of depressive episodes.

A mixed repeated measures analysis of variance revealed significant improvements in self-reported depressive symptoms (IDS-SR) over time for all patients in group therapy (F=17.2, p<0.001, partial eta square=0.18) with a mean of 37.6 at T1, 32.6 at T2, and 29.9 at T3, all scores remaining within the moderate level of symptom severity at the end of group therapy. Although no significant differences are found between bipolar and unipolar patients with regards to their trajectories of improvement, closer observation of the means suggests a trend for unipolar patients to report higher depressive severity scores than bipolar patients across the three measurement periods. Furthermore, after controlling for total number of depressive episodes and comorbid diagnoses, bipolar patients trended towards greater improvement in depressive symptoms compared with unipolar patients while the interaction effect is marginally significant (F=2.86, p=0.06, partial eta square=0.04, **Figure 2**). Results also reveal a significant interaction between depressive symptoms over time and number of comorbid diagnoses (F=3.68, p=0.03, partial eta square=0.09).

According to a mixed ANOVA analysis, social functioning (SAS-SR total mean) improved significantly over time with group interventions for all patients (F=8.81, p=0.001, partial Eta square=0.10) with no significant differences between bipolar and unipolar depressed patients. Changes in social functioning over the course of group therapy are positively correlated with changes in self-reported depressive symptoms (Pearson R=0.582, p ≤ 0.01) for all patients. In addition to the overall mean for social functioning, all patients significantly improved in areas of SAS-work role (F=6.53, p=0.002, partial Eta square=0.08) and SAS-social leisure activities (F=14.1; p=0.001; partial Eta square=0.15) with bipolar patients engaging in significantly more social leisure activities than unipolar patients (F_interaction_=5.54; p=0.005; partial Eta square=0.07). Group therapy treatments contributed as well to significantly lowering perceived stress (PSS; F=12.87, p=0.001, partial Eta square=0.14) with a post-treatment mean for the entire sample (M=23, SD=6.4) only slightly above rates reported for a large non-psychiatric sample in the US (M=22, SD=6.3) (56).

All patients also significantly increased their use of problem-solving coping strategies (CISS; F=6.18, p ≤ 0.003, partial Eta square=0.07), lowered their emotion-oriented strategies (CISS; F=8.69, p=0.001, partial Eta square=0.10), and increased use of social diversion (CISS; F=10.27; p=0.001; partial Eta square=0.12) over distraction as a preferred form of avoidance-oriented coping strategies. Bipolar patients used significantly more social diversion compared to unipolar patients (F_interaction_=4.40; p=0.014; partial Eta square=0.05). Post-treatment means obtained for the CISS in this sample are slightly better than means reported for a unipolar depressed sample by McWilliams et al. (57) particularly in the greater use of task-oriented strategies and lower use of emotion-oriented strategies in the current sample. Bipolar patients also show significantly more interpersonal flexibility than unipolar patients, over the course of group therapy, by endorsing the value of having a wider range of interpersonal behaviors related to the group therapy situation (vector length_CSIV; F_interaction_=2.69; p=0.035; partial Eta square=0.08). Bipolar patients also have baseline measures of interpersonal self-efficacy that are more agentic (CSIE-unagentic score; t=2.33, p=0.01; Cohen’s d=0.48) and less submissive (CSIE-HI-nonassertive; t=2.14, p=0.01; Cohen’s d=0.44) than interpersonal self-efficacy reported by unipolar patients.

All patients reported significant Perceived Improvements with their overall state of health (PIQ; F=3.93, p=0.05, partial Eta square=0.05) over the course of group therapy. The subscale of emotional health improved significantly (F=4.40, p=0.04, partial Eta square=0.05) over 20 weeks of group therapy, while the subscale of social relations improved more slowly but not significantly and the subscale of physical health did not appear to change over time for all patients. All Perceived Improvement scores (PIQ) obtained at the end of group therapy are much lower than scores reported for a sample of patients treated in a methadone maintenance program also receiving psychosocial services, in the same city where the current sample is taken from (49).

Repeated measures ANOVAs using the Weekly Journal over all 20 weeks of group therapy show significant improvements in self-reported behavioral activation from one week to the next (F=2.52; p=0.002) for all patients. All patients also reported significantly fewer depressive symptoms experienced the week prior to each group session over the course of group therapy (F=2.30, p=0.006). A mixed-model ANOVA showed significant differences between both diagnostic groups (F=1.93; p=0.025). Results suggest that unipolar patients report more depressive symptoms each week overall. Results also suggest that bipolar patients gain more interpersonal confidence (CSIE) throughout group therapy as indicated by significantly higher rates compared to unipolar patients (F=2.04; p=0.016).

**Figure 3:**
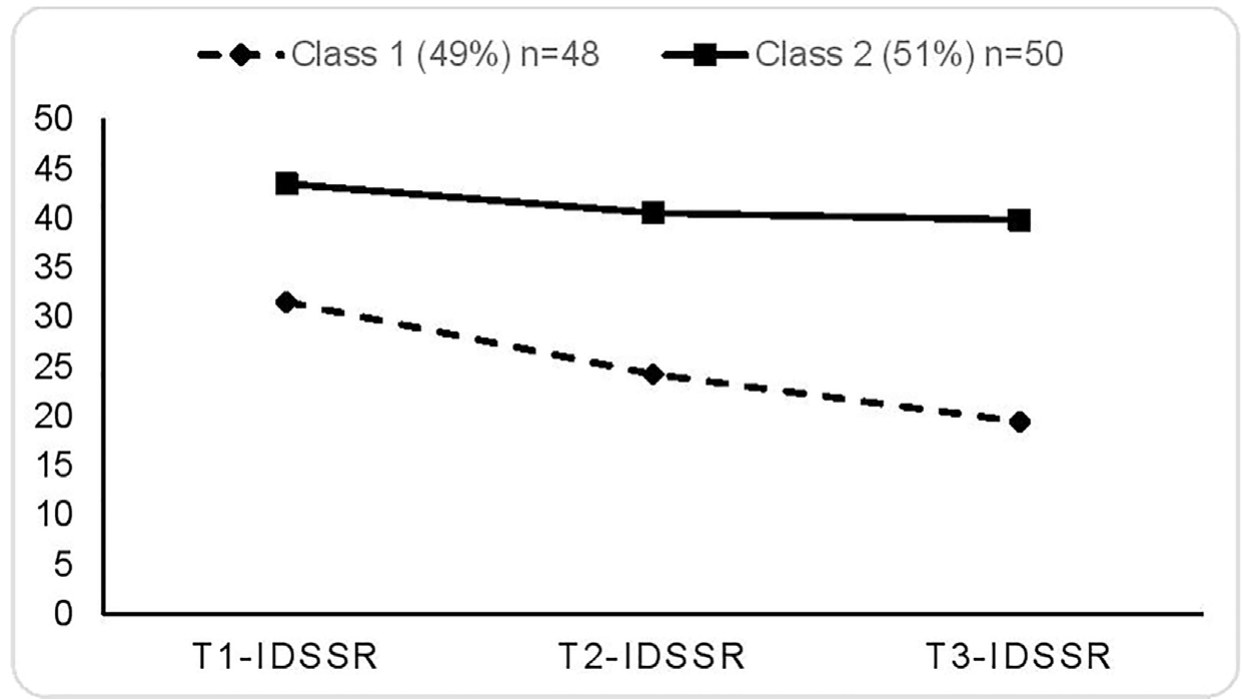
Treatment trajectories for two latent classes of responders and non-responders. Entries are conditional means.

A longitudinal Latent Class Analysis was used to explore underlying classes of trajectories using the self-report measure of symptom severity (IDS-SR) for both samples. Results outlined in **Table 2** demonstrate two latent classes of patients as the most optimal solution: lowest BIC, good entropy, significantly different from the 1-class solution, and not different from a 3-class solution. As shown in **Figure 3**, the first latent class represents moderate to severely depressed patients (49%, mean=32) at the start of group therapy who improved significantly over time, ending group therapy in the mild symptoms range (mean=19). The second latent class represents severely depressed patients (51%, mean=44) at the start of group therapy who did not improve over time (mean=40). A mixed ANOVA confirms significant differences between trajectories of these two latent classes with regards to depressive symptoms (F=16.7; p ≤ 0.001; Partial Eta square=0.17) and almost all baseline social functioning measures (**Tables 3A** **–** **C**). Patients in the first trajectory group who improved significantly over the course of treatment, also improved significantly more in social functioning (overall mean) than patients in the second trajectory group (T= −2,27, p ≤ 0.03, standard error=0.12, 95% confidence).

**Table 2:** Number of optimal latent classes – trajectories of depressive symptoms (IDS-SR).

Parameters		Number of latent classes			
		1	2	3	4
BIC		2,121.6	2,100.5	2,102.7	2,106.2
Entropy		–	0.76	0.72	0.78
LMR adjusted test (p-value)		–	0.004	0.24	0.10

BIC, Bayesian Information criterion; LMR, Lo Mendel Rubin.

**Table 3A:** Latent classes for depressive symptoms trajectories by participant characteristics: frequencies comparison (n=98).

T1-Characteristics	Latent classes		
	Class 1:Low depression with decreasing trajectory	Class 2:High depression with stable trajectory	Chi-2
n	48	50	
Gender			1.96
Female	23 (43)	31 (57)	
Male	25 (57)	19 (43)	
Marital status			
Maried	16 (50)	16 (50)	0.04
Single/divorced	24 (48)	26 (52)	
In a relationship	8 (50)	8 (50)	
Employment status			
Employed	9 (69)	4 (31)	3.51
Unemployed	22 (49)	23 (51)	
Sick leave	14 (40)	21 (60)	
Student/retired	3 (60)	2 (40)	
Diagnosis			0.68
Unipolar	35 (47)	40 (53)	
Bipolar	13 (57)	10 (43)	
Prevalence depression			
3 episodes or more	30 (50)	30 (50)	0.02
2 episodes or less	17 (49)	18 (51)	
Comorbidity			0.41
Yes	19 (45)	23 (55)	
No	29 (52)	27 (48)	

Entries are frequencies (percentages in parenthesis, calculated across 2 latent classes).

**Table 3B:** Latent classes of depressive symptoms trajectories by participant characteristics: Means comparison (n=98).

T1- Characteristics	Latent classes			
	Class 1: Low depression with decreasing trajectory	Class 2: High depression with stable trajectory	t	Cohen’s d
n	48	50		
Age	45.3 (9.41)	46.1 (11.1)	0.43	–
Duration of current episode (months)	27.7 (27.5)	24.0 (16.1)	0.83	–
Total depressive episodes	3.30 (1.52)	3.33 (2.43)	0.09	–
SAS mean	2.33 (0.46)	2.84 (0.51)	5.16***	1.06
Work Role	3.02 (1.42)	3.51 (1.09)	1.88	–
Social leisure	2.70 (0.67)	3.19 (0.65)	3.67***	0.75
Extended family	1.98 (0.54)	2.41 (0.51)	4.05***	0.83
Primary relation	2.15 (0.41)	2.61 (0.66)	3.01**	0.83
Parental role	1.84 (0.66)	2.25 (0.53)	1.80	–
Family unit	1.85 (0.69)	2.51 (1.20)	3.31***	0.68
Vector Length CSIE	3.07 (1.40)	4.08 (1.71)	3.15**	0.66
Vector Length CSIV	1.11 (0.49)	1.32 (0.54)	1.91	–
Vector Length IIP	1.64 (0.78)	2.18 (0.82)	3.26**	0.68
CSIE_BC (dominant-distant)	4.31 (2.10)	3.38 (1.93)	2.27*	0.47
CSIE_NO (dominant-friendly)	5.32 (2.30)	4.36 (2.06)	2.18*	0.45
CSIE Agency_Y	−1.75 (1.90)	−3.05 (1.80)	3.33**	0.71
CSIV mean	2.12 (0.46)	1.88 (0.55)	2.20*	0.47
CSIV_BC (dominant-distant)	1.35 (0.64)	0.91 (0.69)	3.23**	0.67
CSIV raw agentic	2.07 (0.63)	1.75 (0.59)	2.54*	0.53
IIP mean	1.56 (0.52)	1.68 (0.52)	1.12	–
IIP PA (domineering)	0.77 (0.66)	0.51 (0.58)	2.09*	0.43
IIP FG (avoidant)	1.94 (0.90)	2.54 (1.09)	2.98**	0.61
IIP HI (non-assertive)	2.22 (1.01)	2.64 (1.08)	2.11*	0.43
IIP NO (intrusive)	1.17 (0.96)	2.17 (1.04)	2.61**	0.53
IIP raw unagentic	2.15 (0.78)	2.58 (0.84)	2.66**	0.54
IIP Agency_Y	−1.19 (0.82)	1.88 (0.86)	3.98**	0.82

Entries are means (with standard deviation in parenthesis). The Cohen’s statistic is a measure of effect size: 0.20=Small effect; 0.50=Medium effect; 0.80=Large effect. *p ≤ 0.05, **p ≤ 0.01, ***p ≤ 0.001 (two tailed-test).

**Table 3C:** Latent classes of depressive symptoms trajectories by participant characteristics: means comparison (n=98).

T1-Characteristics	Latent classes			
	Class 1:Low depression with decreasing trajectory	Class 2:High depression with stable trajectory	t	Cohen’s d
n	48	50		
CISS coping				
Task oriented	45.65 (10.27)	40.88 (9.70)	2.36*	0.48
Emotion oriented	52.08 (7.61)	54.06 (9.21)	1.16	–
Avoidance oriented	39.44 (8.68)	36.36 (8.01)	1.83	–
Distraction	20.35 (5.23)	19.22 (4.98)	1.10	–
Social Diversion	12.42 (4.04)	10.86 (4.54)	1.79	–
PSS stress	24.83 (5.28)	28.28 (4.88)	3.36***	0.68
T2-Perceived efficacy -PIQ	29.5 (8.51)	24.98 (5.68)	2.89**	0.63
Emotional health	7.93 (2.72)	6.79 (2.12)	2.15*	0.48
Social relations	5.62 (1.85)	4.23 (1.17)	4.14***	0.91
Physical health	6.17 (2.25)	5.47 (1.72)	1.62	–
Weekly Journal				
Behavioral activation	8.45 (6.52)	5.63 (4.77)	2.17*	0.50
Depressive symptoms	9.03 (7.04)	12.92 (8.39)	2.23*	0.51

Entries are means (with standard deviation in parenthesis). The Cohen’s statistic is a measure of effect size: 0.20=Small effect; 0.50=Medium effect; 0.80=Large effect. *p ≤ 0.05, **p ≤ 0.01, ***p ≤ 0.001 (two tailed-test).

None of the baseline demographic variables, including bipolar vs unipolar diagnosis, duration of current depressive episode, total number of depressive episodes, distinguish these two latent classes as apparent in **Tables 3A**, **B**. Patients in trajectory group 2 have a higher rate of comorbid psychiatric diagnoses, although this did not reach significance. These patients also report more baseline problems with social functioning (SAS-SR overall mean), particularly in areas of reduced motivation and interest for social and leisure activities (SAS-social-leisure), withdrawal, avoidance and/or interpersonal conflicts with extended family members (SAS-extended family) and excessive worrying or guilt about one’s current situation (SAS-family unit) over the past two weeks. They report significantly more perceived stress (PSS) over the past month and tend to use significantly fewer task-oriented coping strategies (CISS-task) than patients in the first trajectory group who report improvements with group therapy.

Patients in trajectory group 1 who benefit most from group therapy also report significantly higher rates of Perceived Improvements (PIQ) in overall health, in emotional health and in social relations compared to more severely depressed patients, even after 10 weeks of group therapy. Furthermore, increasingly endorsing the importance of a wider range of interpersonal behaviors (CSIV vector length), over the course of group therapy, was significantly correlated with improvements in emotional health by the end of treatment (Pearson R=0.330, p=0.001). In fact, reported improvements over time in overall health, emotional health and physical health were significantly correlated with lower levels of reported interpersonal problems over time that are associated with a more rigid behavioral repertoire (IIP vector length). This is an important finding considering how resistant to change physical symptoms have proven to be.

The Logistic Regression Analysis (**Table 4**) revealed that, after controlling for other variables, high baseline problems in social functioning (SAS-SR mean) increase the chances of membership in the severely depressed trajectory group 2 (OR =12.6; 95%CI=1.83–86.7). In addition, low rates of Perceived Improvements with treatment (PIQ) by the end of group therapy also increase the likelihood of membership in this more severe trajectory group 2 (OR=0.75; 95%CI=0.65–0.87). Therefore, higher baseline problems in social functioning represent the most important clinical predictor of membership in the second trajectory group of severely depressed patients who do not report improvements in depressive symptoms over the course of group therapy and who perceive their treatment as ineffective.

**Table 4:** Logistic Regression models predicting trajectory group 2 of non-responders.

T1-Predictors	Model 1		Model 2	
	B (se)	OR	B (se)	OR
SAS mean	2.08*** (0.56)	8.00	2.53** (0.98)	12.6
Vector Length CSIE	0.27 (0.21)	0.19	0.31 (0.28)	1.37
Vector Length IIP	0.44 (0.38)	0.25	0.54 (0.58)	1.71
PSS stress	–	–	0.09 (0.08)	1.09
CISS coping	–	–	0.02 (0.04)	1.02
T3- Perceived efficacy PIQ	–	–	−0.29*** (0.08)	0.75
Intercept	−7.23*** (1.67)	0.001	−3.46 (3.56)	0.03
N	91		77	
Nagelkerke R-square	0.38		0.68	
% ranking	74		83	

Entries are logistic regression coefficients (B) with standard errors (se) in parenthesis, and odds ratios (OR). T1= time 1, T3= time 3. **p ≤ .01; ***p ≤ .001.

## Discussion

This is the first pilot study examining the feasibility of CBASP in a group format with bipolar patients currently in a depressive episode, using the same manualized treatment administered to a sample of unipolar depressed patients within the same psychiatric institution. Bipolar and unipolar moderately depressed patients report significant improvements in self-reported depressive symptoms and social functioning with 20 weeks of group psychotherapies, both CBASP and BA. After controlling for baseline differences, bipolar disorder patients trended towards a greater improvement in depressive symptoms. Similar to previous studies (58–60), bipolar disorder patients in this study report significantly fewer depressive symptoms than unipolar patients in their daily functioning the week prior to beginning group therapy. Bipolar patients in this study were mobilized, resorted to more social activities and used social diversion as a coping strategy while increasingly endorsing the importance of widening their repertoire of interpersonal behaviors over the course of CBASP group therapy. Indeed, treatment outcome was more dependent on the severity of the depressive episode at baseline than on diagnosis alone.

Close to half of patients responded to group therapy (49%, mean=19) in this study reporting a mild level of symptom severity after group therapy. These results are comparable to percentages of responders (48%, mean=10 at posttreatment, 12 weeks) reported by the large multi-site Keller et al. (17) study for individually administered CBASP in combination with pharmacotherapy. However, Keller et al. used the clinician-rated Hamilton Rating Scale for Depression [HRSD-24, (61)]. Posttreatment means (M=22) reported by Michalak et al. (25) for 8 sessions of group CBASP added to treatment as usual, using the self-report Beck Depression Inventory [BDI, (62)], are lower than those reported by the entire sample at posttreatment (M=30) in this study using the IDS-SR. However, Michalak et al.’s outcome scores are higher than means for responders to group therapy according to the Latent Class Analysis in this study (M=19). Estimated comparisons of scores on the IDS-SR, the BDI and the HRSD-24 (63) suggest that posttreatment means cited for the Michalak et al. sample and for this study are both in the moderate range of symptom severity at posttreatment. However, responders in this study within the first latent class group ended therapy in the mild level of symptom severity, comparable to HRSD-24 scores reported by Keller et al. (mean=10). Results reported by Sabass et al. (23) for inpatient group CBASP reveal a comparable large effect size (*d*=1.11) at posttreatment for 24 sessions of group CBASP using the BDI-II (64) as is reported for this study sample (eta square=0.18).

Results confirm studies (26) showing that patients in both diagnostic groups with more severe baseline depressive symptoms do not appear to make significant progress (6, 28) with a 20-week psychological intervention concurrent with pharmacotherapy. Similar to Sabass et al.’s findings, this study also finds that patients with a moderate level of depression severity at baseline and therefore fewer problems with social functioning also tend to perceive benefits gained from group therapy. The more severely depressed patients, comparatively, tend to perceive group therapy as ineffective, perhaps related to feelings of defeatism and hopelessness described by McCullough in chronically depressed patients with early trauma. Furthermore, previous research indicates that 12 sessions of group (31, 65) or individually delivered CBASP (20, 66) may not be enough to promote remission in patients with chronic unipolar depression. Miklowitz et al. (7) reported beneficial effects of 30 sessions in 9 months, of intensive psychotherapy for bipolar depression. It would be worthwhile to examine whether more severely depressed patients might benefit more from individually administered CBASP instead of a group format or from prolonged maintenance treatment.

Good baseline social functioning is the most important predictor of reported improvements in depressive symptoms for all patients, with a strong effect size, although post-treatment levels of social functioning remain below levels reported for a non-psychiatric population in all subscales, with work role being the most problematic (42). These results are comparable to those obtained by Michalak et al. who reported no effects with CBASP regarding social functioning. Patients in the current study who respond to group therapy by reporting lower levels of depressive symptoms also make significantly more improvements in social functioning than non-responders. Other studies have shown that functional impairment predicts clinical outcome in unipolar depression (67) and has been used as an outcome measure to classify a sample of bipolar remitted patients into good versus poor functional outcome and then comparing individual characteristics of each group (68). These results also support research on staging of mood disorders pointing to deteriorating social functioning as a contributor to illness progression (26).

These encouraging results regarding group therapy contributing to improved social functioning in moderate to severely depressed patients, underscore the importance of extending the duration of psychosocial interventions for individuals with severe depression knowing that interpersonal changes need more time to consolidate (69). Patients’ reports of Perceived Improvements in overall and physical health, over the course of group therapy in this study, are also related to improvements in their interpersonal problems through the acquisition of a wider range of interpersonal behaviors such as group CBASP promotes with interpersonal problem-solving skills. These results are supported by previous findings of the beneficial impact of psychotherapy in reducing interpersonal problems of depressed individuals (70) and underline the importance of addressing social functioning in psychotherapy for moderate to severe depression.

Results also support previous research that interpersonal dispositions of low agency, social avoidance (71, 72) and a limited repertoire of interpersonal behaviors contribute to the severity of depressive symptoms (73) and treatment non response (74). McCullough (12) describes a similar unagentic profile regarding interpersonal functioning of persistently depressed individuals. Unipolar patients reported significantly more baseline unagentic and submissive interpersonal dispositions than bipolar patients, in this study, which may explain their lower gains in interpersonal self-efficacy over the course of group therapy. Although no differences in baseline social functioning between the two diagnostic groups are observed and unipolar patients reported being significantly more behaviorally active the week prior to the start of group therapy, bipolar patients appear to attribute more value to interpersonal interactions and mobilize themselves towards change over the course of group therapy demonstrating increased interpersonal confidence. These findings need to be replicated with a larger sample of bipolar patients to further explore interpersonal dispositions of bipolar depressed patients.

Findings reported in this pilot study do not support the exclusion of bipolar patients in a depressive episode from treatment with CBASP for moderate to severe depression. The perception of bipolar depression as being difficult to treat may be a result of the higher medical and psychiatric comorbidities with Bipolar Disorders. However, according to these preliminary results, this perception seems to be unwarranted with regards to providing CBASP in a group format to bipolar depressed outpatients. Indeed, this study suggests that bipolar patients in a depressive episode can benefit as much from the same psychological treatment provided to unipolar patients with chronic depression. No assertion is made as to the recommendation of using CBASP to treat Bipolar Disorders. Rather, CBASP addresses the social withdrawal, interpersonal difficulties and cognitive distortions associated with a severe depressive episode observed in both diagnostic groups. These findings suggest that when controlling for psychiatric comorbidities and number of depressive episodes, perhaps bipolar depressed patients might benefit even more form group CBASP compared to unipolar depressed patients. This study merits to be repeated with a larger sample of bipolar patients currently in a depressive episode. Perhaps bipolar depressed patients can join unipolar depressed patients in group CBASP together and benefit from sharing their similar and different characteristics.

## Limitations

This pilot study is the first to document treatment benefits with group CBASP for bipolar outpatients currently in a depressive episode, using the same manualized group CBASP administered to unipolar depressed patients. Its strength is in its prospective nature, the inclusion of moderately to severely depressed patients and in an extended 20-week treatment duration (instead of the shorter, 12-week duration previously reported as insufficient) provided under similar conditions and following similar procedures for all patients. Limitations of the study include a relatively small sample of bipolar patients, however comparable (n ≤ 100) to other bipolar psychotherapy studies (7, 9). Another limitation is the use of only self-report measures of improvements in depressive symptoms and social functioning. Adding clinician-rated measures of improvements provides a more objective measure of change that is known to be different from subjective measures. This study did not assess patients at a follow-up period for possible deterioration in mood or in gains achieved and did not use a control or comparison group. Following this pilot study, future research objectives need to demonstrate the effectiveness of group CBASP for unipolar and bipolar depression in a randomized controlled study using a comparison group. Longer treatment duration, including maintenance sessions and long-term follow-up may benefit the more severely depressed patients and is also recommended. Offering the more severely depressed patients individual sessions after group therapy may also help address early trauma or social anxiety that require further interventions.

## Data Availability Statement

The raw data supporting the conclusions of this article will be made available by the authors, without undue reservation.

## Ethics Statement

The studies involving human participants were reviewed and approved by: The Douglas Mental Health University Institute’s Research Ethics Board (REB Protocol 10/19). The patients/participants provided their written informed consent to participate in this study.

## Author Contributions

LS is the principal investigator responsible for design, conceptualization, and research objectives, and played a leading role in all drafts of the manuscript. ET is the statistician responsible for all data analyses, synthesizing results, and presentation of tables and figures. EF had a substantial role in data acquisition: carried out all data organization and entry into SPSS and again in REDCap software. SB is the medical consultant on design, methodology, and discussion of results, and revision and editing of manuscript. SRen is a medical consultant and made contributions in presentation of results. SRej was involved in writing, reviewing, and editing the manuscript, and contributed to interpretation of data and design/presentation of analyses. MP played a substantial role in conception and design of statistical analyses. All authors revised the work critically for important intellectual content, gave final approval of the version to be published, and agree to be accountable for all aspects of the work in ensuring that questions related to the accuracy or integrity of any part of the work were appropriately investigated and resolved.

## Conflict of Interest

LS: Co-author of two Group CBASP manuals with small royalties received from Taylor and Francis publishers in 2016. 1. Group Treatment Manual for Persistent Depression: Cognitive Behavioral Analysis System of Psychotherapy (CBASP) Therapist’s Guide (2016) 2. Group Workbook for Treatment of Persistent Depression: Cognitive Behavioral Analysis System of Psychotherapy-(CBASP) Patient’s Guide (2016). SB: Peer-reviewed research funding: CIHR, Pfizer Research Award, Narsad Research support, kt and contract: Lundbeck, Otsuka, Sunovion Consultant-advisory board: Allergan, Janssen-Ortho, Lundbeck, Otsuka, Sunovion Speaker bureau: Janssen-Ortho, Lundbeck, Otsuka, Sunovion Stock holdings/patents: None Community organization: Board member of REVIVRE (Pro Bono). SRej Fonds de Recherche Québec Santé (FRQS) Junior Investigator Award: Receives salary support from Satellite Healthcare: Received an investigator-initiated research grant for an unrelated project. SRen Sponsored conferences by: Janssen, Purdue Pharma Advisory boards or similar committees in the pharmaceutical industry: Janssen, Lundbeck, Otsuka, Canada.

The remaining authors declare that the research was conducted in the absence of any commercial or financial relationships that could be construed as a potential conflict of interest.
